# Family affluence and cultural capital as indicators of social inequalities in adolescent’s eating behaviours: a population-based survey

**DOI:** 10.1186/1471-2458-12-1036

**Published:** 2012-11-28

**Authors:** Anne-Siri Fismen, Oddrun Samdal, Torbjørn Torsheim

**Affiliations:** 1 Department of health promotion and development, University of Bergen, Christiesgate 13, Bergen, 5015, Norway; 2 Department of psychosocial science, University of Bergen, Christiesgate 12, Bergen, 5015, Norway

**Keywords:** Social inequality, FAS, Material capital, Cultural capital, Adolescents, Healthy eating, Fruit, Vegetables, Sweets, Soft drinks, Meal frequency

## Abstract

**Background:**

Dietary inequality, via socio-economic inequality, may involve several mechanisms. Different aspects of adolescents’ socio-economic circumstances should therefore be considered in order to make effective interventions to promote healthy eating in the young population. Indicators designed to tap socio-economic status among adolescents in particular will facilitate a better understanding of the concept of socio-economic status and how it influences health behaviour among young people. The purpose of this study was to evaluate if material capital and cultural capital individually and independently contribute to the prediction of eating habits in the Norwegian adolescent population.

**Methods:**

The analysis is based on survey data from the Health Behaviour in School-Aged Children study. The Family Affluence Scale (number of cars, holidays, PC and bedrooms) and number of books in the household were used as indicators of socio-economic status, respectively measuring material capital and cultural capital. Their influence on adolescent’s consumption of fruit, vegetables, sweets, soft drinks, and consumption of breakfast and dinner was evaluated. Pearson’s correlation, logistic regression and ridit transformation analysis were used to analyse the data.

**Results:**

Higher family affluence was shown to predict consumption of more fruit (OR 1.52) and vegetables (OR 1.39) and consumption of breakfast (OR 1.61) and dinner (1.35). Cultural capital was significantly associated to consumption of fruit (OR 1.85), vegetables (OR 2.38) sweets (OR .45), sugary soft drinks (OR .26), breakfast (OR 2.13) and dinner (OR 1.54). Cultural capital was the strongest predictor to healthy eating among adolescents in Norway.

**Conclusions:**

Material capital and cultural capital individually and independently contributed to the prediction of healthy eating patterns among adolescents in Norway. Cultural capital is an understudied dimension of the socio-economic status concept and the influence on health behaviour needs to be explored in future studies. Initiatives to promote healthy eating should focus on education, habits and consciousness of a healthy diet, but also at reducing the high cost of fruit and vegetables. There is further a need for developing appropriate indicators for adolescent socio-economic status.

## Background

Among the large number of young people in Norway who not does follow national dietary guidelines [1], those from families with low socio-economic status (SES) are at particular risk. Their diet is likely to contain less fruit and vegetables [2,3] and more sweets and soft drinks [4,5], and the frequency of their meals is likely to be less regular [6], than for their counterparts from families with high SES. Socio-economic variations in diet are of concern because they are associated with a range of health conditions and therefore represent pathways by which socio-economic inequalities lead to inequalities in health status [7]. Adolescents, in particular, are a group to target strategically when aiming to reduce observed disparities in food habits from a global public health perspective. Adolescence is the most important period for establishing food preferences and habits that will be maintained into adulthood [6,8], and it is the transition period during which familial disadvantages are forming the basis for young people’s SES as independent adults [9,10].

Relatively little is known about the mechanisms underlying socio-economic disparities in eating habits or how to offset inequalities among young people. The Nordic public health service has accordingly acknowledged the need for further research on socio-economic mechanisms determining variations in adolescents’ diet [11]. Identifying distinct aetiological pathways between socio-economic status and eating habits would contribute valuable knowledge to policymakers and others who need to design interventions that effectively reduce social inequalities in diet.

Dietary inequality, via SES inequality, may involve several mechanisms. Education, occupation and income are most frequently used as indicators to explain variations in SES [3]. Education may influence food habits by facilitating or constraining reading and comprehension of nutritional information and compliance with nutritional recommendations as well as influence values attached to healthy eating [12,13]. Occupation may affect diet through role modelling in work-based relations and social networks [14,15]. Income may reflect the availability of economic and material resources, which could directly determine dietary quality by making healthy food more affordable and readily accessible to those with higher incomes. Also cultural capital has been linked to health inequalities [16,17]. Cultural capital can be broadly defined as people’s symbolic and informational resources [18]. These resources, which are varying across the social classes and status groups [19], have been suggested to influence health behaviour through the operational skills, knowledge, health considerations and norms acquired through education and socialization [16].

Each of the above approaches contributes to a complex understanding of the SES construct. However, some researchers seem to assume that different SES indicators reflect the same underlying information and can therefore be used interchangeably [20]. This assumption is reflected in studies using only one indicator even when several are available, as well as in studies making indices without adjusting for the unmeasured effect of one indicator on another [21,22]. The assumption that different indicators can be used as proxies for the same phenomenon is challenged by the results of studies that examine correlations between different SES indicators. Typically, correlations between education, occupation and income are weak to moderate (magnitude 0.3–0.6) in developed countries [23,24]. This finding suggests some shared associations but also, more importantly, highlights the unique contribution of each indicator. It also supports the view that the different indicators are conceptually distinct, and that their influences on eating habits are transmitted through social processes. Additionally, the relevance of a specific indicator might differ between subgroups of the population, such as between adults and adolescents. In a study of a US sample, Krieger et al. [25] found that different SES indicators differed between gender, ages and different ethnicities. This underscores the need for specific knowledge in order to target intervention approaches according to age, gender and ethnicity. Moreover, one dimension of SES may be of particular relevance in promoting healthy behaviours such as consuming fruits and vegetables, while another dimension may play a significant role in promoting health-compromising behaviours such as consuming food rich in fat and sugar. Differential association can be viewed as a methodological issue but is also strongly relevant to operating mechanisms, since differential associations across SES indicators may signal specificity in the processes influencing eating behaviours [20,26].

Further complicating the issue of specificity of indicators is the difficulty adolescents have in reporting their parents’ education and occupation. Even more serious are the systematic missing data from adolescent respondents from families with low SES, who frequently experience difficulties in reporting parental SES [27]. As a supplement to conventional indicators, the above limitations call for proxy indicators tailored to the socio-economic circumstances of adolescent populations. Against this background, SES-based differences in adolescents’ consumption of fruit, vegetables, sweets and sugary soft drinks and in meal frequency were in the current study investigated among Norwegian students at junior high school (age 11, 13 and 15) and senior high school (age 16) using two different dimensions of SES: Material capital measured by the Family Affluence Scale (FAS) [28], and cultural capital measured by number of books in the household. The effect of material resources is evaluated as an important determinant of food choices in adult populations, but little is known about how such resources affect eating patterns in adolescent populations. The economic situation of families in Nordic countries is of particular importance for policymakers trying to facilitate healthy food choices by adolescents, because fruit and vegetables are quite expensive. The FAS taps young people’s absolute socio-economic status based on material markers, provides an alternative to the more traditional social class [27,28], and is conceptually related to common consumption indices of material deprivation [29] and home affluence [27]. Cultural capital has thus far been rarely studied as a dimension of SES in relation to inequalities in diet [17,30]. In the present study, Bourdieu’s [31] concepts of cultural capital and habitus are used as an approach to the formation of taste and consumption, which, among other initiatives, is expected to influence young people’s attitudes towards healthy eating. The concept of cultural capital refers to social abilities and competence for action, including perceptions, behavioural norms and operational skills that are needed to deal effectively with health issues on an everyday basis [16]. Habitus consists of previously established categories of perception that create an inner felt understanding of what is valuable and what is not [32]. According to Bourdieu [31], books represent an objectivized form of cultural capital, and number of books in the family has previously been used as an indicator of cultural capital in several inequality studies among children and adolescents [32-38]. Number of books in the household may be seen to act as an indicator of the underlying cultural capital [32] and is likely to be positively associated with education.

To our knowledge, cultural capital as a determinant of eating habits among adolescents has never been studied in a national, representative sample. Our aim was to evaluate the predictive values of material capital (family affluence) and cultural capital as determinants of eating habits among adolescents in Norway.

## Methods

In this study, data from participants in the Norwegian study of the World Health Organization (WHO) cross-national HBSC study (2005/2006) were used. The sample was selected using a stratified standard cluster sampling procedure to ensure national representativeness [39]. The primary sampling unit was the school class, with one participating class per age group. The school was used if school class information was not available. The sample was randomly selected using a standard cluster sampling procedure based on a geographical stratified list and sequentially selection from a randomized starting point. Of the 551 invited schools 378 chose to participate in the study. Data were collected from 3,318 boys and 3,129 girls from sixth (mean age: 11.5), eighth (mean age: 13.5) and tenth (mean age: 15.5) grade junior high school and from first grade senior high school (mean age: 16.5). In the participating schools the student response rate was 85% at age 11, 86% at age 13, 85% at age 15 and 81% at age 16. Non-responses comprised primarily either class- or school-level non-participation or student absence on the day the survey was administered.

Participation was based on parental passive consent. The students answered the internationally developed, self-administered HBSC questionnaire in the classroom after receiving standardized instruction from their teacher [39]. The data collection was anonymous. The students were asked not to report their names on the questionnaire and to return the completed questionnaire in a provided sealed envelope, to ensure that only the researchers had access to this information. The Privacy Ombudsman at the Norwegian Social Science Services assured that the study complied with privacy and confidentiality requirements. The study was sent to the Norwegian Western Regional Ethical Committee and was evaluated to not need ethical clearance.

### Questionnaire

The questionnaire was developed through international consensus and translated into the languages of participating countries. The Norwegian questionnaire was pilot tested. To ensure that the translation communicated the intended connotations and concepts, an independent re-translation back into English was approved by independent researchers in the international study [39].

### Socio-economic status

#### The family affluence scale (FAS)

The FAS is a measure of material affluence derived from the characteristics of the household. It consists of the following four items: 1. “Does your family own a car, van or truck?” (No = 0, One = 1, Two or more = 2). This item is a component of the Scottish deprivation index developed by Carstairs and Morris [29], which is used widely in health inequalities research. **2.** “How many times did you travel away on holiday with your family during the past 12 months?” (Never = 0, Once = 1, Twice = 2, Three or more times = 3). This item is a measure of “deprivation of home facilities” [40]. **3.** “Do you have a bedroom for yourself?” (No = 0, Yes = 1). This item is a proxy for overcrowding, classified by Townsend [40] as housing deprivation, and is also a component of the Scottish deprivation index. **4.** “How many computers do your family own?” (None = 0, One = 1, Two = 2, More than two = 3). This item was introduced into the FAS to differentiate among SES groups in affluent countries [41]. These FAS items provide a scale that can be easily completed by the students.

#### Cultural capital

Cultural capital was measured by the number of books in the student’s home. The following question was asked: “How many books are there in your family? (Usually there are 40 books on each row of the bookshelf)” (None = 0, 1–10 = 1, 11–50 = 2, 51–100 = 3, 101–250 = 4, 251–500 = 5). Evans et al. [34] showed that this item has a high test-retest reliability.

#### Transformation of SES variables

A central assumption of the present study is that the FAS and number of books have at least ordinal measurement properties, and that the scores of the scales can be used to rank individuals and groups along a latent continuum of material wealth and cultural capital. Ridit transformation is a method of analysis for such ordinal variables that proceeds from the assumption that the ordered categorical variable is an approximation to an underlying, but not measurable, continuous variable [42]. Ridit transformation is a widely used approach for SES scales with ordinal measurement [43,44], also in previous HBSC studies [45]. Ridit transformation converts ordered categorical responses to cumulative probabilities. In the present study, the FAS and number of books were ridit transformed to yield a continuous material deprivation score or a continuous cultural deprivation score, respectively, ranging from 0 to 1, with a whole-sample mean of 0.5. The material deprivation score reflects the proportion of adolescents with a higher level of family affluence. The cultural deprivation score reflects the proportion of adolescents with a higher number of books in the household. A student with a deprivation score of 1 is at the bottom of the material/cultural hierarchy (100% of the other students have a higher level of material/cultural capital), whereas a student with a score of 0 is at the top of the material hierarchy (no other student has a higher level of affluence). In the predictive model with different eating habits as dependent variables, the regression coefficient of the material/cultural deprivation score can be directly interpreted as the predicted difference in eating habits between the least deprived individual and the most deprived individual. This valuable property has been exploited in a series of studies using ordinal SES ratings [43,44,46].

### Eating habits

#### Fruit, vegetables, sweets and soft drinks

Consumption of fruit, vegetables, sweets and sugary soft drinks were measured by one item for each frequency: “How many times a week do you eat fruit/vegetables/sweets/sugary soft drinks?” (Never = 0, Less than once a week = 1, Once a week = 2, Two to four times a week = 3, Five to six times a week = 4, Once a day = 5, More than once a day = 6). Students eating fruit and vegetables at least once daily were identified by the responses: “Once a day” and “More than once a day”. This is in line with recommendations for a healthy lifestyle having fruit and vegetables as a part of the everyday diet.

#### Meal frequency

Meal frequency was measured by the following questions: “How often do you usually eat breakfast?” and “How often do you usually eat dinner?” (Every day = 1, Four to six times a week = 2, One to three times a week = 3, Rarely or never = 4). Students eating breakfast or dinner every day were identified by the response “Every day.” Breakfast and dinner are meals provided by the family on an everyday basis. Lunch was not included in this study, because lunch is a meal mostly eaten outside the family context.

## Results

### Consumption of food and meal frequency among adolescents

Table 1 presents the percentages and numbers of boys and girls by intake of fruit, vegetables, sweets and sugary soft drinks and by meal frequency. In general, girls consumed fruit and vegetables more often and soft drinks less often than did boys. The gender differences were less consistent with regard to meal frequency. In the group of 15-year-olds, boys were more likely to eat a daily breakfast and dinner than were girls. Among the whole study cohort, the older students consumed less fruit and vegetables and more sweets and sugary soft drinks than did the younger students. The older students were also more likely to miss breakfast.

**Table 1:** Percentage (and n) of boys and girls reporting daily consumption

	**Fruit**	**Vegetables**	**Sweets**	**Soft drinks**	**Breakfast**	**Dinner**
11 y boys	42.4% (331)	30.7% (287)	4.4% (34)	9.2% (71)	83.2% (664)	81.7% (627)
11 y girls	56.5% (449)	40.0% (316)	5.7% (45)	5.7% (45)	80.7% (636)	86.7% (680)
Gender diff ^χ2^	p < .001	p < .001	ns	p < .01	ns	p < .01
13 y boys	34.7 (287)	27.4% (226)	9.2% (76)	14.1% (117)	76.2% (632)	87.4% (726)
13 y girls	44.7% (337)	30.5% (229)	9.6% (72)	11.0% (83)	65.9% (494)	82.9% (622)
Gender diff^χ2^	p < .001	ns	ns	p < .05	p < .001	p < .01
15 y boys	31.6% (257)	22.6% (184)	12.9% (105)	21.0% (171)	66.4% (539)	84.7% (689)
15 y girls	40.7% (291)	29.7% (212)	13.1% (94)	13.7% (98)	51.6% (368)	77.6% (553)
Gender diff^χ2^	p < .001	p < .01	ns	p < .001	p < .001	p < .001
16 y boys	21.4% (186)	17.5% (152)	11.1% (97)	22.5% (196)	60.1% (525)	84.7% (689)
16 y girls	38.5% (228)	29.2% (249)	15.4% (131)	14.0% (119)	55.3% (471)	78.8% (670)
Gender diff^χ2^	p < .001	p < .001	p < .01	p < .001	ns	p < .01
Grade diff ^χ2^	p < .001	p < .001	p < .001	p < .001	p < .001	ns

χ^2^: chi-square.

### Frequency of SES indicators

We found no statistically significant gender differences in the FAS. Girls reported having more books at home than did boys (p < .001). Chi-square analysis confirmed that older students reported higher socio-economic status than did younger students (p < .001).

### Socio-economic status and eating habits

We tested the hypothesis that material capital and cultural capital individually and independently contribute to the prediction of eating habits. A Pearson’s correlation analysis of the two SES variables disproved the null hypothesis (r = .20) but did not indicate the relative importance of each factor to eating habits. The second step of the data analysis used logistic regression to examine the independent contribution of each SES indicator to eating habits. Age and gender were included as covariates in the logistic regression analysis. Changes in NagelKerke from block 1 to 2 in the model was .053 - .069 = .016 for fruits, .028 - .037 =.09 for vegetables, .028 - .049 =.021 for sweets, .040 - .068 =.028 for soft drinks, .070 - .090= .020 for breakfast and .006 – .013 = .007 for dinner. As shown in Table 2 and Table 3, statistically significant associations were found between cultural capital and daily intake of respectively fruit (odds ratio, OR, of 1.85), vegetables (OR 2.38), sweets (OR .45), soft drinks (OR .26), breakfast (OR 2.13) and dinner (OR 1.54). The association between FAS and eating habits was statistically significant for fruit (OR 1.52), vegetables (OR 1.39), breakfast (OR 1.61) and dinner (OR 1.35). We found no statistically significant association between FAS and respectively sweets and soft drinks. Cultural capital was shown to be the strongest independent predictor of all outcomes.

**Table 2:** The likelihood of material capital (FAS) and cultural capital (number of books) associated with daily consumption of fruit, vegetables, sweets and soft drinks

**Food items**	**Fruit (n=6058)**		**Vegetables (n=6051)**		**Sweets (n=6049)**		**Soft drinks (n=6052)**	
	**OR**	**95% CI**	**OR**	**95% CI**	**OR**	**95% CI**	**OR**	**95% CI**
Material capital (FAS)	1.52	(1.25–1.82)	1.39	(1.12–1.69)	1.14	(.83–1.54)	1.12	(.85–1.47)
Cultural capital (number of books)	1.85	(1.52–2.22)	2.38	(1.92–2.94)	.45	(.33–.61)	.26	(.17–.34)

OR Odds Ratio.

CI Confidence Interval.

**Table 3:** The likelihood of material capital (FAS) and cultural capital (number of bookds) associated with meal frequency

**Meal frequency**	**Breakfast (6047)**		**Dinner (6040)**	
	**OR**	**95% CI**	**OR**	**95% CI**
Material capital (FAS)	1.61	(1.32–1.96)	1.35	(1.05–1.72)
Cultural capital (number of books)	2.13	(1.72–2.63)	1.54	(1.2–1.96)

OR Odds Ratio.

CI Confidence Interval.

## Discussion

Our findings indicate that cultural capital was a stronger predictor than material capital of disparities in consumption of fruit and vegetables and in the regular eating of breakfast and dinner, and it was the only significant predictor of consumption of sweets and sugared soft drinks among young people in Norway. The analyses support the argument that cultural capital and material capital are distinct dimensions of SES that work through different mechanisms to make unique and separate contributions to health. These findings accord with other studies using number of books in the household and FAS [47-50] as SES indicators in research on eating habits among adolescents. For example, in a regional sample of Norwegian adolescents, Iversen and Holsen [48] found that students with high SES reported consuming more fruit and vegetables and less soft drink than did their counterparts with low SES, and that the number of books in the household was more strongly associated with eating habits than was family affluence. Vereecken and colleagues found a more regular meal pattern among adolescents in groups with higher family affluence [50].

Bourdieu’s concepts of cultural capital and habitus link structural and behavioural determinants of health by explaining how people’s behavioural options and preferences are related to lifestyle [30]. This approach encompasses both the conscious choice of health promoting behavior and the often milieu specific more or less routine patterns of perceptions, and suggests that adolescent’s food habits are influenced by parental perception and attitudes to food habits. Acquisition of cultural resources depends on the social class-specific learning context [51], and the family milieu represents a particularly important arena for learning eating habits during childhood and adolescence. Values attached to health, and knowledge about health effects of certain food choices, are class-specific cultural resources that structure people’s references and choices [30]. It is likely that students living in families with high cultural capital learn how to eat healthy at home. This perspective is supported by Farakas and Hibel [52], who analyzed several aspects of the home environment and concluded that home library size had a strong predictive validity as an indicator of parents’ attraction to the teaching role vis á vis their children. Parental social support is indeed shown to be more strongly associated with adolescent food habits than is social support from friends and social groups [53,54]. Also habitual processes are at work here, and the family’s habits and norms for what kind of eating patterns are considered appropriate and required, are developed through this socio-cultural context. Adolescents living in a family context of regular meals and where diets high in fruit and vegetables and low in sugar and fat are desirable as well as accessible and available, are likely to develop healthy eating habits consistent with the collective lifestyle of the family.

Further economic circumstances influence a family’s opportunity to buy healthy food. The lower frequency of fruit and vegetable consumption among adolescents from families with low SES may reflect the high cost of fruit and vegetables in Norway. Focus groups, surveys and interviews have repeatedly shown that the relatively high cost of fruit and vegetables is a barrier to healthy eating for people with low incomes [55-59]. Material capital is a resource that enables families to buy healthy food. High-cost food such as fruit and vegetables might therefore be less accessible for adolescents in families with low SES. However, economical capital approaches often fall short in elucidating the social differences observed in those health behaviours that cannot be explained by financial determination: unhealthy patterns of consumption that in large parts are determined by people’s norms and values [30]*.* When people are operating within a given economic frame of options, they also seem to bring cultural resources into play. Dibsdall and colleagues [60] showed that a barrier to increased consumption of fruit and vegetables in groups with low SES is not only the higher cost but also the group members’ preference for less healthy food items. Brug and colleagues [61] showed that a positive preference for fruit and vegetables increases the likelihood of their daily consumption. They also showed that taste preferences mediate differences in fruit and vegetable consumption between genders. Øygard [62] argued that food preferences differ between social groups, and that expensive food has a symbolic value that can be used to represent higher cultural capital, a perspective supported by Abel [16]. These points may explain our finding that the FAS, representing material capital, is an important predictor of consumption of fruit and vegetables but not of sweets and soft drinks, while number of books, representing cultural capital, is the strongest predictor of food preferences and meal frequency. Norms, values, health consciousness and habits attached to cultural capital therefore seem more important than economic factors with respect to healthy food choices.

### Limitations

The validity of adolescent SES is challenged conceptually and methodologically. The current study may be criticized for its lack of conceptual saturation in using the number of books in the household as a measure of cultural capital. However, number of books is considered a suitable item when assessing cultural home environment among adolescents and widely used in inequality research in this age group [32-38]. Number of books may work as a proxy for education, but one can also argue that books might tap a broader dimension than education. Moreover, books in the family might work above and beyond formal education, especially among the younger population that not yet has started their own educational carrier. Use of this indicator could be further criticized in light of trends toward a minimalistic lifestyle in contemporary societies, characterized by having few items (including books) in the living room and by increased access to e-books. On the other hand, e-books were not widespread among Norwegian families in 2005/2006 when these data were collected and have probably not replaced the family’s printed books. Girls reported having more books at home than boys did (p <.001). One possible explanation to this is that girls differ from boys in their perceptions of books at home, and gender differences must then be taken into account when using number of books as an indicator of cultural capital.

The FAS was developed not to tap material capital among Norwegian adolescents in particular but rather to investigate socio-economic circumstances among young people in Europe and North America [47]. Computers are currently used in Norwegian school settings and for daily homework. Most teenagers therefore have their own computer. Low automobile ownership might reflect not low SES but rather the environmental consciousness of parents. However, the FAS is shown as a valid instrument to measure material circumstances among young people and currently used to this purpose. Concerning the validity of reported food habits, the Health Behaviour in School-aged Children (HBSC) questionnaire for measuring intake of fruit, vegetables, sweets and soft drinks has been recognized as a valid instrument in epidemiological studies ranking adolescents according to their usual food intake [63]. Finally, our findings and conclusions might be relatively more applicable for a comparatively egalitarian society such as Norway compared to other countries where economic factors might play a relatively bigger role for food choices.

### Conclusions and implications

The identification of cultural capital and material capital as independent and significant predictors should be welcomed as a valuable basis for health-promoting programmes aiming to increase healthy eating habits among adolescents in families with low SES. Differences in cultural capital point to the relevance of policy initiatives promoting healthy purchasing habits, nutritional knowledge and social support for healthy eating, particularly for adolescents with low SES. The present study indicates that home milieu is of great importance with regard to young people’s dietary habits, but also schools should be considered as a vital setting for learning – and improving – eating habits. The school milieu constitutes a core part of children and adolescents every day socio-cultural context, and thereby important role modeling behaviour for healthy eating may take place. Further, as school is an arena where all SES groups can be reached, interventions aiming to promote healthy eating for all students have the potential to reduce SES differences in dietary behavior. In Norway, school based educational programs, with both a theoretical component that focuses on the benefits of healthy eating and a practical component that teaches young people how to prepare a meal, have been trialed. Unfortunately, this intervention had no observed effect on participants’ consumption of fruit and vegetables. Bere et al. [2] suggested that the intervention did not succeed in changing the participants’ preferences because it did not change their access to fruit and vegetables at school. In order to make fruit and vegetables more accessible for all SES groups, the Norwegian government in 2007 decided to offer a programme fully subsidizing fruit and vegetables in junior high schools (grades 8–10) and combined schools (grades 1–10). The effect of this initiative is so far contradictory [64,65].

Changes to the taxation and welfare systems are other strategies for addressing differences in SES. Reducing the price of fruit and vegetables sold in schools and limiting the availability of sweets and soft drinks during school time are initiatives that directly influence adolescents’ food habits. Several studies have shown that when prices were reduced by 50%, purchases of fruit increased three- or fourfold [66,67], while purchases of vegetables increased twofold [66]. Initiatives, such as reducing the price of healthy food, can be implemented through policy-making, and they could increase the total consumption of fruit and vegetables. However, groups with high SES tend to profit most from such health-promoting interventions, since they already have a healthy diet consciousness [2,68].

The finding that cultural capital and material capital are independent SES indicators is also important with regard to some of the current practices and conceptual assumptions underlying the use of SES indicators in research on eating habits. This finding underscores the importance of researchers providing an explicit rationale or justification for their choice of a particular SES indicator. It is important to recognize that different SES indicators may provide different, and therefore complementary, information, and that, generally, no one indicator is superior to any other [43]. Studies based on the assumption of interchangeable SES indicators may not give a complete picture and may misrepresent the degree of dietary differences between SES groups. In order to achieve healthy eating patterns among all adolescents, and particularly among those in families with low SES, a more comprehensive understanding of inequalities in food consumption is required. Future research should focus on developing SES indicators that can tap the variations in access to educational and cultural resources among adolescents. A concrete example could be further development of the family affluence scale in order to take the socioeconomic context in countries with high welfare into account.

## Abbreviations

FAS: Family affluence scale; HBSC: Health Behaviour in School-aged Children; OR: Odds ratio; CI: Confidence interval; SES: Socio-economic status; WHO: World Health Organization; χ2: Chi-square.

## Competing interests

The authors have no competing interests.

## Authors’ contributions

All the authors contributed to the design of the study. ASF and TT analysed the data and ASF drafted the first version of the manuscript. All authors have contributed to interpretations of the findings and provided amendments to the text. All authors read and approved the final manuscript.

## Pre-publication history

The pre-publication history for this paper can be accessed here:

http://www.biomedcentral.com/1471-2458/12/1036/prepub
